# Depressive Symptoms and HIV Viral Suppression: A Systematic Review and Meta-analysis

**DOI:** 10.1007/s10461-024-04571-0

**Published:** 2024-12-18

**Authors:** Bishan Huang, Alitasha Younger, Mary P. Gallant, Thomas J. O’Grady

**Affiliations:** 1 School of Public Health, University at Albany, State University of New York, Rensselaer, NY USA; 2 New York State Department of Health, AIDS Institute, Albany, NY USA; 3 School of Public Health, Downstate Health Sciences University, State University of New York, New York City, NY USA; 4 Zuckerberg College of Health Sciences, University of Massachusetts Lowell, Lowell, MA USA

**Keywords:** Depression, HIV viral suppression, PLWH, Meta-analysis, PRISMA

## Abstract

Previous research suggests that depression impacts HIV outcomes, including viral suppression. This meta-analysis quantifies the association between depression and HIV viral suppression. A systematic literature search was conducted in PubMed, Web of Science, EBSCO, and OVID to identify studies published through 2012 to 2022. The software Rayyan was used to evaluate eligibility of studies, and the 2020 Preferred Reporting Items for Systematic Reviews and Meta-analysis guidelines were used for abstracting data. A random effects meta-analysis was performed using Review Manager 5.4.1. Of the 1911 articles screened, 16 studies were included covering 80,103 participants. The results showed individuals without depression were more likely to achieve HIV viral suppression or undetectable viral load compared to individuals with depression (OR 1.30; 95%CI 1.15, 1.48; I^2^ = 76%). Subgroup analysis indicated this effect was significant among the general population of people living with HIV (n = 75,353; OR 1.32; 95%CI 1.12, 1.55; I^2^ = 85%) and cisgender women living with HIV (n = 4553; OR 1.28; 95%CI 1.09, 1.50; I^2^ = 16%), but not among cisgender men living with HIV (most identified as men who have sex with men) (n = 197; OR 2.13; 95%CI 0.43, 10.61; I^2^ = 83%). This meta-analysis shows a significant positive association between the absence of depression and achieving HIV viral suppression overall and among the subgroup of cisgender women. Public health interventions for people living with HIV should include strategies to identify and address the depressive symptoms that impact adherence to treatment, increase the risk of psycho-behavioral co-morbidities, and exacerbate social or structural factors impeding viral suppression.

## Introduction

Viral suppression is crucial to HIV prognosis and management. Even with treatment, people living with HIV (PLWH) with unsuppressed viral loads have a higher risk of disease progression, transmission, and mortality [1–3].Viral suppression is typically defined as having less than 200 copies of HIV per milliliter blood [4]. In 2021, approximately 74 to 78% PLWH in the U.S. received some HIV care, and 65 to 68% PLWH were virally suppressed [4]. The “Ending the HIV Epidemic in the U.S.” initiative aims to increase the percentage of people with diagnosed HIV who are virally suppressed to at least 95% by 2025 and remain at 95% by 2030 [5]. HIV infections often co-occur with substance abuse and mental disorders [6]. Despite the drastic decrease in HIV incidence in recent years, HIV still disproportionately affects sexual and racial minority groups and people who inject drugs [7]. Among PLWH, the estimated depression rate is two-to-four times higher than that of the general population [8–12].

Previous research has established an association between depressive symptoms and HIV viral non-suppression [13–15]. Additionally, a recent review highlighted the complex effects of social determinants of health on the association between depression and HIV outcomes, underscoring the need for further clarification [6]. Depression was associated with higher HIV viral loads, lower CD4 cells counts, hastened progression to AIDS, reduced adherence to antiretroviral therapy (ART), compromised medication outcomes, and elevated risks of mortality [13, 16–19]. Evidence also suggests that depression may impair killer lymphocyte activity and lead to increased activated CD8 T lymphocytes and viral loads among HIV infected women [20]. Therefore, the link between depressive symptoms and unsuppressed viral loads was supported by both decreased treatment adherence and biomedical pathways. Depressive symptoms increase the likelihood of unsuppressed viral loads even among individuals receiving ART [21]. However, the strength and characteristics of the relationship between depression and HIV viral suppression are unclear.

While depression interventions for PLWH led to (or were associated with) increased ART adherence [15, 22, 23], limited evidence is available regarding the direct interventional effects on HIV viral load. Understanding the strength and mechanism of the relationship is essential for resource allocation, intervention design, and policy approaches to improve HIV outcomes in the U.S. It will also be valuable for guiding cost-effectiveness analysis of depression interventions for PLWH and integrating mental health services into HIV care to advance the progress in ending the HIV epidemic in the U.S. [6, 24, 25].

To build upon and update previous reviews regarding the role of depression in HIV disease progression [6, 26, 27], this systematic review and meta-analysis aims to comprehensively investigate the relationship between depression and HIV viral suppression, as well as to quantify and characterize this relationship.

## Methods

This study aligns with the 2020 Preferred Reporting Items for Systematic Reviews and Meta-analysis (PRISMA) guidelines [28]. Researchers used the systematic review software Rayyan to select eligible studies [29]. Due to the nature of the study design, an Institutional Review Board (IRB) review is not required.

### Search Strategy and Inclusion/Exclusion Criteria

A literature search was conducted on PubMed, EBSCO, Ovid, Web of Science, and Google Scholar using the following search terms in titles and abstracts: [(HIV) OR (AIDS) OR (Human Immunodeficiency Virus)] AND [(depression) OR (depressive symptom)] AND [(viral load suppression) OR (viral load) OR (viral suppression) OR (load suppression)]. The search covered articles published from January 1, 2012 to December 31, 2022. The language of the literature was restricted to English.

Studies were included if they met all the following criteria: The study assessed the association between depression or depressive symptoms and HIV viral load suppression or detectable viral load in people living with HIV/AIDS; the study presented quantitative results of the association in the outcomes; the study was conducted in the United States or Canada; it was an empirical study and published in a peer-reviewed journal; it was published in English; it was published between January 1, 2012 and December 31, 2022. Studies were excluded from the review if they met any of the following criteria: The study was a review, comment, study protocol, non-peer-reviewed or a non-empirical report; the study population was non-human subjects; the study did not clearly present quantitative results regarding the main research question; the study was conducted outside of the United States or Canada; the study was not published between January 1, 2012 and December 31, 2022; the study did not provide sufficient quantitative data for the meta-analysis.

### Data Extraction and Quality Assessment

Eligible studies were reviewed by two qualified researchers independently and the following data were abstracted: (1) first author’s name; (2) year of publication; (3) country where the study was conducted; (4) study design; (5) study population; (6) sample size; (7) measure of depression; (8) measure of HIV viral load suppression/non-suppression or detectable/undetectable viral load; (9) outcomes; (10) conclusion. Disagreement in data extraction was resolved through discussion.

Two researchers independently examined the quality of the selected articles using the Methodological Index for Non-Randomized Studies (MINORS) [30], which is appropriate for assessing cross-sectional and cohort studies. The MINORS scoring system allocates a score of 0 to 2 for each of the 12 items according to whether the item was reported and whether it was treated adequately [30]. The items are scored 0 (not reported), 1 (reported but inadequate), or 2 (reported and adequate). The global ideal score is 16 for non-comparative studies and 24 for comparative studies. To minimize the effects from the items not applicable to some studies, researchers used the mean score for quality evaluation (0–1.32: low; 1.33–1.66: medium; > 1.66: high). Discrepancies in scoring were resolved by discussion. See Appendix for more details of the data extraction and quality assessment processes.

### Data Analysis

Outcomes for which data could not be directly compared across studies were synthesized qualitatively. The thematic analysis approach [31] was used to synthesize the most relevant qualitative characteristics of the study outcomes and discussions about those outcomes. Researchers identified themes from the outcome and discussion sections from each of the included articles and selected the most relevant information to be incorporated into the current study.

Quantitative outcomes were synthesized from the studies when sufficient and comparable data were available. The pooled prevalence of depression or depressive symptoms were presented as percentages. The dichotomous outcome measure was HIV viral load suppression versus non-suppression, or detectable versus undetectable viral load. A meta-analysis was performed using Review Manager 5.4.1 [32]. Results were presented as odds ratios and Forest Plots.

Heterogeneity among the studies was assessed using the I^2^ test [33]. Heterogeneity was defined as absent when I^2^ was between 0 and 25%; low, between 25.1% and 50%; moderate, between 50.1 and 75%; or high, between 75.1 and 100%. A fixed-effects model was used in the meta-analysis when p ≥ 0.1 and I^2^ ≤ 50%; otherwise, a random-effects model was used [33]. Subgroup analysis (meta-regression) was also performed to test sensitivity of the pooled estimate to study characteristics (i.e., study population, study design, etc.) given that heterogeneity was high [33].

### Publication Bias

To assess publication bias, the researchers plotted each study’s estimated OR against the standard error of the estimate to generate a funnel plot [34]. Asymmetry in the plot could potentially indicate that studies with small, statistically non-significant estimates were not submitted or accepted for publication [34]. Egger’s regression test was used to further assess publication bias with an intercept value of 0 indicating no publication bias and a p-value smaller than 0.1 indicating statistical significance of the value [35, 36].

## Results

### Search Results

**Fig. 1:**
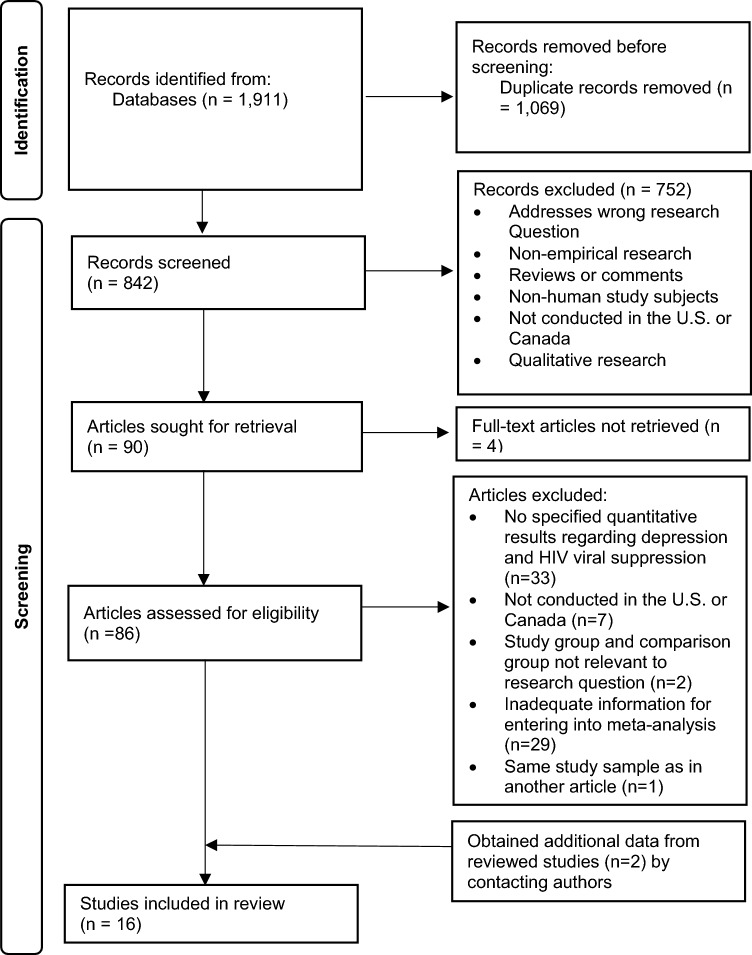
Flow chart detailing the search for and selection of studies

The systemic search identified 1911 potentially eligible studies, and no additional records were found during manual searches of reference lists. After removing 1069 duplicates using Rayyan, another 752 studies were removed during the screening of titles and abstracts. Of the remaining 90 articles, four could not be retrieved from the internet nor through interlibrary loan. A total of 86 articles were included in full-text review. After selecting articles based on full-text review, we retrieved additional data for two eligible articles through contacting the authors. In the end, 16 articles were selected for the systematic review and meta-analysis after excluding ineligible articles. See Fig. 1 for details.

### Characteristics of Included Studies

The 16 selected studies include six cross-sectional [37–42] and ten longitudinal cohort studies [43–52], altogether involving 80,103 participants. Of the participants, 46,473 (58.0%) were non-depressed and 33,630 (42.0%) were depressed. Sample sizes ranged from 81 to 52,300, with a mean of 8432.0, a median of 973, and a standard deviation of 18,639.0 [41, 42]. Eleven studies involved a clinical sample [37–44, 47, 48, 50], whereas five studies had participants from both clinical and non-clinical settings [45, 46, 49, 51, 52]. Six studies focused on cisgender women living with HIV [37, 45, 49–52], among which one focused on pregnant women [50]; Two studies focused on cisgender men living with HIV, most of whom were men who have sex with men (MSM) [38, 41]; and eight studies were on the general PLWH of different gender identities and sexual orientations [39, 40, 42–44, 46–48]. Four studies focused specifically on youth (aged 18–30) living with HIV [38, 40, 41, 46] and the rest focused on adults of all ages. Each of the longitudinal studies was based on secondary data analyses of existing large cohort surveys while most of the cross-sectional studies involved primary data collection. Seven studies used chart review or administrative data as a complementary data source [37–40, 42, 44, 50]. Fifteen studies were conducted in the United States [37–42, 44–52] and one was conducted in Canada [43]. See Tables 1 and 2 for more details.

**Table 1:** Characteristics of studies identified from systematic review (sorted alphabetically by first author)

Study	Study design	Source population	Independent variable	Dependent variable
Aibibula et al. (2018) [43]	Longitudinal	HIV–HCV co-infected people in Canada initiated in 2003 (data collected from 2012 to 2015)	Depressive symptoms (CES-D-10, cut-off = 10)	Detectable viral load (≥ 50 copies/ml)
Anderson et al. (2018) [37]	Cross-sectional	Adult women attending an HIV-specialty clinic (2014–2015)	Depression (CES-D, cut-off = 16)	Detectable viral load [> 20 copies/ml]
Castel et al. (2016) [44]	Longitudinal	HIV-infected persons enrolled at the 13 DC Cohort study clinical sites (January 2011 to June 2014)	Mental health/depression (ICD9 coding)	Viral suppression: achieving VS (viral load < 200 copies/ml); sustaining VS and time to virologic failure (VL ≥ 200 copies/ml after achievement of VS)
Chowdhury et al. (2019) [38]	Cross-sectional series (trend analysis)	Young Black men within the Medical Monitoring Project cohort—adults who received clinical care for HIV in U.S. (2009–2015)	Depression (PHQ-8, cut-off = 10)	Undetectable viral load (< 200 copies/ml in the past 12 months)
Gokhale et al. (2019) [42]	Cross-sectional series	Medical Monitoring Project cohort (2009–2014)	Depression, defined as documentation of depression on either outpatient or inpatient medical record abstraction from the patient’s main source of outpatient HIV clinical care)	Sustained viral suppression (all viral loads in past year < 200 copies/ml)
Jain et al. (2021) [39]	Cross-sectional	Racially diverse, indigent patients living with HIV (PLWH) who were obtaining care in an urban safety-net hospital system and had completed a Patient Health Questionnaire-9 (PHQ-9) in 2014 or 2015	VS (highest viral load obtained in the selected year < 200 copies/ml)	Depression (PHQ9, cutoff = 10)
Kassaye et al. (2019) [45]	Longitudinal	The WIHS cohort, a prospective cohort study of HIV-positive women with semiannual study visits and a minimum of 5 follow-up visits was conducted from 1994 to 2017	Depression (CES-D, cut-off = 16)	Intermediate to High probability of viremia (Viremia defined as HIV viral load level greater than 200 copies/ml)
Kohn et al. (2021) [46]	Longitudinal	Youth living with HIV aged 18–24 years with behaviorally-acquired HIV infection and no prior ART except limited ART to prevent mother-to-child HIV transmission	Depressive symptoms (Beck Depression Inventory total scores ≥ 14 at baseline)	HIV non-suppression defined as having either detectable virus at study completion, or inconsistent suppression across the study (detectable virus at ≥ 2 visits) < 200 copies”
Lesko et al. (2021) [47]	Cohort	Johns Hopkins HIV Clinical Cohort (JHHCC). The JHHCC includes all patients who enroll in care in the Johns Hopkins HIV Clinic and who consent to share their data (> 90% of patients)	Current depression in the absence of treatment (PHQ8, cutoff = 10)	Viral non-suppression (> 200 copies/ml)
Li et al. (2020) [48]	Longitudinal	All PLWH who enrolled in the Medical Care Coordination program from January 1, 2013 through September 31, 2017	Depressive symptoms PHQ-9, cut-off = 10)	Viral suppression (< 200 copies/ml) indicated by the most recent VS measurement within 12 months prior to MCC enrollment
McFall et al. (2013) [49]	Cohort	HIV-infected women participating in the Women’s Interagency HIV Study (WIHS) from April 2006 to March 2011	Depression (CES-D, cut-off = 16)	Virologic failure (> 200 copies/ml)
Momplaisir et al. (2018) [50]	Cohort	Women living with HIV who had a live delivery in Philadelphia (2005–2013) and enrolled in the PCM program during pregnancy	Depression diagnosis during pregnancy	Viral suppression at delivery (< 200 copies/ml)
Shacham et al. (2017) [40]	Cross-sectional	Patients aged 18–30 years who received care at the Washington University HIV Clinic in St. Louis, Missouri, throughout 2013	Depressive symptoms (PHQ-9)	Unsuppressed viral load (< 200 copies/ml)
Solomon et al. (2020) [41]	Cross-sectional	A convenience sample of 81 young black MSM recruited from the pediatric/adolescent clinic of the Grady Infectious Disease Program clinic in Atlanta, GA, from November 2015 to July 2016	Depressive symptoms (CESD-R, 20 items, cut-off = 16)	Detectable viral load (> 40 copies/ml)
Spence et al. (2021) [51]	Cohort	Women living with HIV from the metropolitan Washington, DC site of the WIHS (2017–2018)	Depression (CES-D, cut-off = 16)	Viral suppression (< 200 copies/ml)
Wilson et al. (2018) [52]	Longitudinal	Participants drawn from the WIHS cohort (2014–2016)	Depression (CES-D, cut-off = 16)	Viral suppression (< 200 copies/ml across two visits)

**Table 2:** Findings from studies identified from systematic review (sorted alphabetically by first author)

Study	Relevant findings
Aibibula et al. (2018) [43]	People experiencing depressive symptoms had 1.32 times (95%CI 1.07, 1.63) the risk of having detectable HIV viral load, but had comparable CD4 count to people who did not experience depressive symptoms (fold change of CD4 = 0.96, 95%CI 0.91, 1.03)
Anderson et al. (2018) [37]	Depression was not significantly associated with detectable viral load (bivariate OR 0.974, 95%CI 0.492, 1.929, p = 0.94; adjusted OR 0.642, 95%CI 0.268, 1.537, p = 0.32)
Castel et al. (2016) [44]	Mental Health/Depression was not significantly associated with achieving VS [HR = 1.03(0.96, 1.10); aHR = 1.00(0.93, 1.07)]. But Mental Health/Depression was significantly associated with earlier time to virologic failure [HR = 1.47(1.28, 1.70); aHR = 1.24(1.06, 1.45)]
Chowdhury et al. (2019) [38]	Depression was not associated with VS (Rao-Scott chi-square test showed no significance)
Gokhale et al. (2019) [42]	Compared to those without depression, those with a depression diagnosis were less likely to achieve sustained viral suppression (aPR 0.95, CI 0.93–0.97); this association held for persons with treated depression compared to no depression (aPR 0.96, CI 0.94–0.99) and untreated depression compared to no depression (aPR 0.92, CI 0.89–0.96)
Jain et al. (2021) [39]	Patients with AIDS who had not achieved HIV VS had higher odds of experiencing depression [aOR 1.81 (95%CI 1.30, 2.54)] with an interaction found between AIDS and HIV VS (p = 0.02)
Kassaye et al. (2019) [45]	Factors associated with high probability of viremia included younger age (odds ratio [OR] 0.99; 95%CI 0.98–0.99; p = .03), African American race (odds ratio [OR] 2.43; 95%CI 1.75–3.37), p < .001), Hispanic race/ethnicity (OR 1.50; 95%CI 1.03–2.19; p = .04), increased levels of depressive symptoms (OR 1.17; 95%CI 1.01–1.36; p = .03), drug use (OR 1.23; 95%CI 1.01–1.51; p = .04), lower CD4+ T-lymphocyte counts (OR, 95%CI 0.82; 0.80–0.85; p < .001), and unstable housing (OR 1.25, 95%CI 1.03–1.50; p = .02)
Kohn et al. (2021) [46]	Adjusted for ART adherence, odds of HIV non-suppression did not significantly differ by group (odds ratio 0.22, [0.04,1.23]); however, greater somatic symptoms at study entry were associated with increased risk of non-suppression over time (odds ratio 2.33 [1.07,5.68])
Lesko et al. (2021) [47]	History of depression (adjusted risk difference [aRD] relative to no history = 5.9%, 95% confidence interval [CI] 1.5%, 10.3%) and current depression (symptoms or diagnosis) in the absence of treatment (aRD relative to no current depression or depression treatment = 4.8%, 95%CI 1.8%, 7.8%) were associated with higher risk of viral non-suppression than no history of depression. Depression treatment mitigated this association (aRD = − 0.4%, 95%CI − 2.5%, 1.7%)
Li et al. (2020) [48]	Patients with a PHQ-9 score ≥ 10 were less likely (40.5%) to be virally suppressed at enrollment (p = .025) than those with lower scores (42.8% for scores 1–9 and 46.0% for scores of 0). By 6 months post-enrollment, those who reported no comorbidities had a significantly greater VS probability than those who reported using stimulants only, housing instability only, high PHQ-9 only, and all three comorbidities. By 36 months post-enrollment, those with a high PHQ-9 increased to a similar VS probability level as those who reported no comorbidities
McFall et al. (2013) [49]	Depression was associated with virologic failure [HR: 1.64, 95%CI (1.35, 2.00)]
Momplaisir et al. (2018) [50]	Depression was associated with VS at delivery: AOR 1, 95%CI (0.6–1.7); depression was associated with VS at 1 year postpartum AOR 1, 95%CI (0.6–1.7)
Shacham et al. (2017) [40]	Moderate to severe depressive symptoms (PHQ-9 score > 15) increased the odds of having unsuppressed viral loads (OR 3.56, 95%CI 1.26,10.07, p < 0.001)
Solomon et al. (2020) [41]	The odds of having a detectable VL differed across the two categories of depressive symptoms. Specifically, detectable VL was significantly higher in participants with significant depressive symptoms than in those without depressive symptoms (aOR = 4.23; 95%CI 1.52–11.72, p < 0.05)
Spence et al. (2021) [51]	Depressive symptoms were not significantly associated with viral suppression (OR 0.99, 95%CI 0.46,2.17, p = 0.9962)
Wilson et al. (2018) [52]	Being below depressive symptom threshold (CES-D < 16) increased the chance of viral suppression (OR 1.37, 95%CI 0.97,1.91, p = 0.07), but the result is not statistically significant

The mean MINORS quality scores for the 16 studies ranged from 1.25 (low) to 1.80 (high) of a possible 2.0. Table 3 describes the quality of the selected articles. Most of the studies (75%) were of medium to high quality, while four studies (25%) were ranked low quality. As was indicated in Table 3, most of the medium-to-high quality studies suggested a significant and positive association between non-depression and HIV viral suppression, whereas the low-quality studies mostly suggested a non-significant association. Most of the studies measured depression/depressive symptoms with a validated scale (e.g., CESD, PHQ-9, Beck Depression Inventory) with corresponding cut-off points. One study measured depression based on clinical records from chart reviews [42]. Most of the studies measured viral suppression using a cut-off point of 200 RNA copies/ml, while three studies measured undetectable viral load using a cut-off of 20 or 40 RNA copies/ml [37, 41, 43].

**Table 3:** Quality assessment (Methodological Index For Non-Randomized Studies) results from studies identified from systematic review

Study	General criteria								Additional criteria for comparative studies				Average	Quality		
	1	2	3	4	5	6	7	8	9	10	11	12	Score*	Ranking	Weight (%)	Odds ratio (95%CI)
Aibibula et al. (2018) [43]	2	2	2	2	0	2	2	0	NA	NA	NA	NA	1.50	Medium	7.1	1.38 (1.01,1.90)
Anderson et al. (2018) [37]	2	1	2	2	NA	NA	NA	0	NA	NA	NA	NA	1.40	Medium	2.7	0.97 (0.49, 1.93)
Castel et al. (2016) [44]	2	1	2	2	0	2	1	0	NA	NA	NA	NA	1.25	Low	7.1	0.79 (0.58,1.09)
Chowdhury et al. (2019) [38]	2	2	2	2	NA	NA	NA	1	NA	NA	NA	NA	1.80	High	1.5	0.95 (0.36, 2.49)
Gokhale et al. (2019) [42]	2	2	2	2	0	NA	NA	0	NA	NA	NA	NA	1.33	Medium	12.9	1.16 (1.11, 1.20)
Jain et al. (2021) [39]	2	2	1	2	NA	NA	NA	0	NA	NA	NA	NA	1.40	Medium	11.2	1.55 (1.35, 1.78)
Kassaye et al. (2019) [45]	2	2	2	2	0	2	1	0	NA	NA	NA	NA	1.38	Medium	9.9	1.50 (1.23, 1.82)
Kohn et al. (2021) [46]	2	1	2	2	0	2	1	0	2	2	1	2	1.42	Medium	2.2	1.03 (0.48, 2.22)
Lesko et al. (2021) [47]	2	1	2	2	0	2	1	0	2	2	2	2	1.50	Medium	8.2	1.68 (1.29, 2.19)
Li et al. (2020) [48]	2	2	2	2	0	2	0	0	NA	NA	NA	NA	1.25	Low	11.8	1.13 (1.02, 1.26)
McFall et al. (2013) [49]	2	1	2	2	0	2	1	1	NA	NA	NA	NA	1.38	Medium	7.9	1.22 (0.92, 1.61)
Momplaisir et al. (2018) [50]	2	2	2	2	0	2	0	0	NA	NA	NA	NA	1.25	Low	4.8	0.89 (0.57, 1.41)
Shacham et al. (2017) [40]	2	2	2	2	NA	NA	NA	0	NA	NA	NA	NA	1.60	Medium	2.5	5.05 (2.46, 10.36)
Solomon et al. (2020) [41]	2	1	2	2	NA	NA	NA	1	NA	NA	NA	NA	1.60	Medium	1.4	4.86 (1.79, 13.18)
Spence et al. (2021) [51]	2	1	2	2	0	2	1	0	NA	NA	NA	NA	1.25	Low	2.2	1.00 (0.46, 2.17)
Wilson et al. (2018) [52]	2	2	2	2	0	2	1	0	NA	NA	NA	NA	1.38	Medium	6.7	1.37 (0.98, 1.92)

The Methodological Index For Non-Randomized Studies (MINORS) scale includes criteria to evaluate the following: (1) A clearly stated aim; (2) Inclusion of consecutive patients; (3) Prospective collection of data; (4) Endpoints appropriate to the aim of the study; (5) Unbiased assessment of the study endpoint; (6) Follow-up period appropriate to the aim of the study; (7) Loss to follow up less than 5%; (8) Prospective calculation of the study size; (9) An adequate control group; (10) Contemporary groups; (11) Baseline equivalence of groups; (12) Adequate statistical analyses. Criteria 9 to 12 are additional criteria in the case of comparative studies

*The items are scored 0 (not reported), 1 (reported but inadequate), 2 (reported and adequate), NA (not applicable). The average score is ranked: Low quality (0–1.32); Medium quality (1.33–1.66); High quality (1.66–2.00)

### Publication Bias

**Fig. 2:**
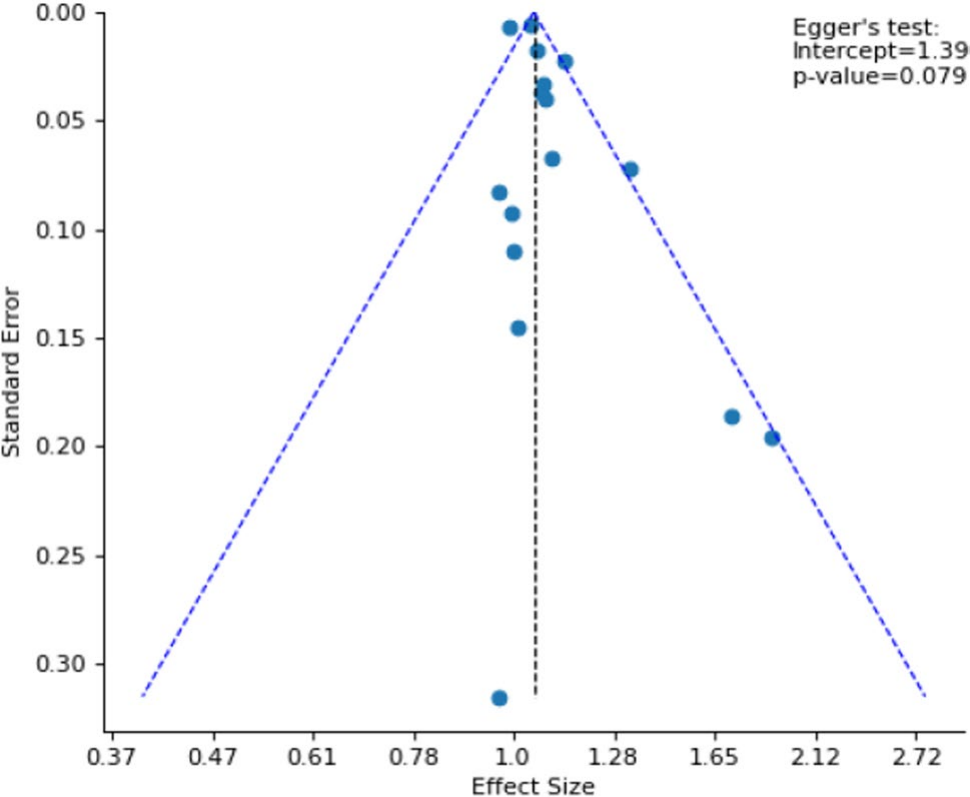
Funnel plot of the effect sizes (ORs) by standard errors of the selected studies and results from Egger’s test

Publication bias exists according to the asymmetric funnel plot (Fig. 2). Most of the effect size data points are clustered near the mean while studies with smaller sample sizes and larger standard errors spread wider at the bottom. This could indicate that studies with a smaller sample size and with non-significant results might not have been published. Significant results from Egger’s regression test (Fig. 2) further confirmed the possibility of publication bias (intercept = 1.39, p = 0.079), indicating that the smaller studies systematically had smaller effect sizes compared to the larger studies and that the asymmetry in the funnel plot did not occur by chance. A p-value smaller than 0.1 was used to indicate statistical significance given that heterogeneity in the studies can hinder the power of Egger’s test [35, 36].

### Prevalence of Depression

The prevalence of depression or depressive symptoms ranged from 9.0% [47] to 60.2% [50] with a mean prevalence of 33.8% and a standard deviation of 14.1%. The mean prevalence was higher in cisgender women living with HIV (mean = 37.9%, SD = 12.0%) and cisgender men living with HIV (mean = 32.9%, SD = 19.8%) but lower in the general population of PLWH (mean = 30.9%, SD = 14.2%) according to the study sample.

### Effects of Depression on HIV Viral Suppression

**Fig. 3:**
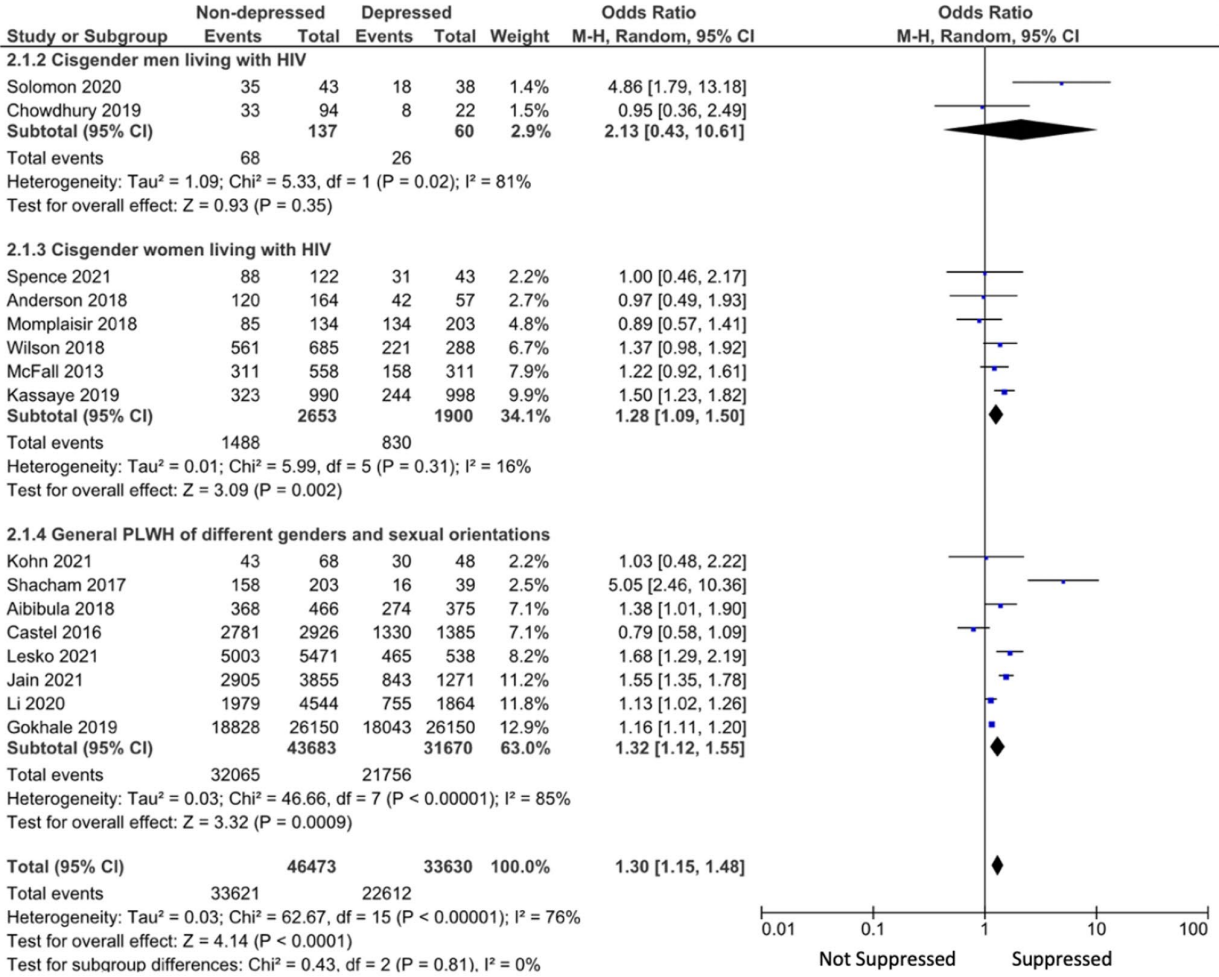
HIV viral suppression among PLWH without depressive symptoms compared to those with depressive symptoms: Results from random effect meta-analysis by study population

Pooled results from the meta-analysis indicated non-depressed PLWH were more likely to achieve viral suppression or an undetectable viral load (OR 1.30, 95%CI 1.15, 1.48) compared to those with depression or depressive symptoms. Depression or depressive symptoms decreased the odds of HIV viral suppression or undetectable viral loads (OR 0.77, 95%CI 0.68, 0.87). Tests for heterogeneity showed positive results (Tau^2^ = 0.03; Chi^2^ = 62.67, df = 15, p < 0.00001, I^2^ = 76%) with a significant overall effect (Z = 4.14, p < 0.0001). See Fig. 3 for details.

The association between depression and HIV viral suppression varied by how HIV viral suppression was measured. Aibibula et al. (2018) identified depressive symptoms as a risk factor for incomplete short-term HIV viral suppression among people co-infected with HIV-HCV (OR 1.32, 95%CI 1.07, 1.63) [43]. Six studies examined sustainable or accumulative HIV viral suppression and had mixed findings [38, 42, 44–46, 52]. For example, Gokhale et al. (2019) showed those with a depression diagnosis had a lower prevalence of achieving a sustained viral suppression (aPR = 0.95, 95%CI 0.93, 0.97) [42]. But Wilson et al. (2018) and Chowdhury et al. (2019) indicated depression was not associated (OR 1.37, 95%CI 0.97, 1.91, p = 0.07; OR 0.95, 95%CI 0.36, 2.49) with viral suppression as an outcome [38, 52].

The effects of depression potentially functioned through decreased ART treatment adherence [38, 44, 46], increased risks for psycho-behavioral co-morbidities [38, 39, 47], and interactions with demographic, psychosocial, or structural factors [41, 49]. For example, Kohn et al. (2021) found that greater ART adherence was associated with decreased risk of viral non-suppression [46]; though the association between depressive symptoms and viral non-suppression extended beyond ART adherence. As an example of the psycho-behavioral co-morbidities, Lesko et al. (2021) found that depression-associated recent use of alcohol, cocaine, and heroin potentially mediated the association between depression and viral non-suppression [47]. Chowdhury et al. (2019) also found those with injection or non-injection drug use had lower durable HIV viral suppression compared to those who did not use drugs (28% v.s. 42%, p < 0.05) [38]. Depression also mediated relationships between sociodemographic factors and viral suppression. For instance, depressive symptoms contributed to racial disparities in viral suppression among women living with HIV [49] and mediated the relationship between housing instability and detectable viral load [41].

Treatment or supportive services targeting depression can impact HIV viral loads. Lesko et al. (2021) found that untreated current depression was associated with higher risk of viral non-suppression (aRD = 4.8%, 95%CI 1.8%, 7.8%), while depression treatment mitigated this association (aRD = − 0.4%, 95%CI − 2.5%, 1.7%) [47]. Gokhale et al. (2019) reported similar results but noted that people with treated depression still had lower prevalence of sustained viral suppression (aPR = 0.96, 95%CI 0.94, 0.99) [42]. Among pregnant women living with HIV, increased clinical engagement during pregnancy mediated the impact of depressive symptoms on viral load status. Additionally, HIV viral suppression outcomes did not differ by depression status, which was potentially due to supportive services received during pregnancy [50].

### Subgroup Analyses

Subgroup analysis (Fig. 3) by study population showed that the association between depression and HIV viral non-suppression was significant among the general PLWH of different genders and sexual orientations (n = 75,353; OR 1.32; 95%CI 1.12, 1.55; I^2^ = 85%) and among cisgender women living with HIV (n = 4553; OR 1.28; 95%CI 1.09, 1.50; I^2^ = 16%), but not among cisgender men living with HIV (n = 197; OR 2.13; 95%CI 0.43, 10.61; I^2^ = 83%). Researchers observed a stronger association in the studies with a predominantly Black or Hispanic sample (n = 13,296; OR 1.39; 95%CI 1.02, 1.89; I^2^ = 76%) compared to those with a racially diverse sample (n = 66,807; OR 1.28; 95%CI 1.13, 1.45; I^2^ = 78%). A stronger association was also detected among the cross-sectional studies (n = 58,086; OR 1.61; 95%CI 1.18, 2.21; I^2^ = 88%) compared to the longitudinal studies (n = 22,017; OR 1.22; 95%CI 1.06, 1.41; I^2^ = 59%). When grouped by outcome definition, studies measuring HIV viral suppression (RNA < 200 copies/ml) showed a significant pooled outcome (n = 78,960; OR 1.28; 95%CI 1.13, 1.46; I^2^ = 78%), whereas studies measuring undetectable viral load (RNA < 20, 40, or 50 copies/ml) showed insignificant results (n = 1143; OR 1.67; 95%CI 0.84, 3.32; I^2^ = 72%).

## Discussion

We performed a comprehensive systematic review and meta-analysis to evaluate the association between depressive symptoms and HIV viral suppression. Overall, ten prospective longitudinal cohort studies and six cross-sectional studies were considered relevant, most of which were judged to be of moderate to high quality. The results from the 16 studies included in the meta-analysis support the hypothesis that PLWH without depression had better HIV viral suppression outcomes, while depression increased the risk of HIV viral non-suppression. These results were consistent with previous studies conducted in the U.S. and other countries [13, 14, 17–19]. The association between depression and HIV non-suppression is further supported by biological evidence, which suggests that depression might alter the activities of killer lymphocytes and consequently lead to increased HIV viral loads [20]. Depressed patients were shown to have immune dysregulations involving alterations in cellular and humoral immunity, which may cause increased susceptibility to immune-related diseases [53–56].

Our analysis revealed a wide range of depressive symptom prevalence among PLWH, with higher prevalence found in cisgender women and cisgender men (most identified as MSM), which align with the results from previous studies [53, 57–59]. Higher prevalence of psycho-social risk factors such as substance use, intimate partner violence, and poor housing status that co-occur with HIV among sexual minority groups may contribute to the higher depression prevalence [60, 61]. Interestingly, we found a stronger association between depression and HIV viral suppression rates in the cross-sectional studies than in the longitudinal studies. This may suggest that individuals with HIV may develop coping strategies or receive interventions that mitigate the impact of depression on HIV management, which is more observable in longitudinal designs. The longitudinal studies can account for confounding variables and change over time, whereas the cross-sectional studies might overestimate associations due to the variability in how depression or HIV viral suppression is measured at a single time point. The results also suggest that depression was diagnosed more systematically in studies that were specifically designed to detect it. If this were true, the association between depression and low viral load rates could be stronger than indicated in this meta-analysis making this an important area of interest for future studies.

The association between depression and viral suppression was significant for viral suppression (RNA < 200 copies/ml) but not for undetectable viral loads (RNA < 20, 40, or 50 copies/ml). This finding highlights the multifactorial nature of depressive symptoms' impact on HIV viral loads at lower levels. For instance, individuals achieving undetectable levels of HIV viral loads might be receiving more comprehensive care or support, which could have mitigated the impact of depression. After achieving viral suppression at a very low level by maintaining strict adherence to treatment, the chance to reach undetectable viral loads might depend more on the characteristics of the individual’s immune system and HIV reservoir [62] but less affected by depression. Further investigation into what factors may impact individuals with depression from achieving undetectable viral loads is warranted.

Our analysis suggests that the association between depression and HIV viral non-suppression was only significant in cisgender women living with HIV and the general population of PLWH (including people of different genders and sexual orientations) but not among cisgender men (most identified as MSM) living with HIV. This contrasts with some previously published research [63, 64]. Small sample sizes might be responsible for the decreased capability to detect significant results in the studies of cisgender men. For instance, Solomon et al. (2020) showed strong statistical significance with a sample of MSM but Chowdhury et al. (2019) did not show significant results with a sample of 72% MSM and 28% other cisgender men [38, 41]. These findings suggest that MSM and other cisgender men may have different outcomes, warranting further investigation into the unique factors affecting depression and HIV suppression in sexual and gender minority groups. Further investigation is warranted to examine the unique factors associated with depression and HIV viral suppression in sexual and gender minority groups.

Given the biological and behavioral pathways linking depression to viral suppression, there are several policy implications that stem from the results of this meta-analysis. Our findings suggest HIV management would benefit from including screening and follow-up interventions for depression. Previous research in Uganda showed cost-effectiveness of depression screening and antidepressant therapy with fluoxetine at ART initiation or re-initiation in reducing loss to follow-up and increasing viral suppression rates [25]. A study comparing different intervention methods found group support psychotherapy to be more cost-effective in averting disability-adjusted life-year in PLWH [24]. Further studies can use our findings as a foundation to examine the cost-effectiveness of different types of depression interventions on improving HIV outcomes for PLWH in the U.S. In addition, routine and free or low-cost depression screening among PLWH could identify subgroups of PLWH who have or are at risk for depression and in need of follow-up intervention. Because depression could exist at time of diagnosis, or develop over time among PLWH, a standard of care should include periodic depression screenings as part of HIV management. Furthermore, health insurance plans should consider covering depression screenings and follow-up treatments as part of comprehensive HIV care.

This study has several notable strengths. It addresses an important clinical and public health issue and provides an updated look at depression and viral load suppression, adheres to established PRISMA guidelines, uses the MINORS criteria to assess article quality, and the meta-analysis provides greater precision of results than individual studies. Finally, most of the cited studies used validated scales to measure depression and standard measures of viral suppression. However, there are also limitations in our study. None of the studies in the meta-analysis specifically focused on transgender individuals or older PLWH and only one study [50], focused on pregnant women. Transgender women face a significantly higher HIV burden and worse outcomes associated with mental health disorders, higher risk behaviors, physical abuse, social isolation, economic marginalization, and unmet transgender-specific healthcare needs [65, 66]. Older PLWH with comorbidities often have higher rates of depressive symptoms [67]. Pregnant women have higher risk of non-adherence to ART and difficulty utilizing prenatal care if they are depressed, and these will likely lead to adverse outcomes in HIV viral loads [68]. Future studies of PLWH should target a more diverse sample or focus on high-risk sexual/gender minority groups and pregnant women. Further, community samples are under-represented in the meta-analysis; most of the studies had a clinical sample. Further study is warranted to examine whether outcome difference exists between a community versus a clinical sample of PLWH.

Also, this meta-analysis does not include any randomized controlled trials (RCT). Limited RCTs on depression exist for PLWH, and only a few focus on viral suppression outcomes. The four RCTs we identified during abstract screening did not meet the inclusion criteria for the meta-analysis [69–72]. More RCTs are needed to further examine the effectiveness of depression interventions on HIV viral suppression for PLWH. RCTs or longitudinal cohort studies are also needed to establish and validate the dose–response relationship between depression and viral non-suppression to better guide the use of intervention resources. Future studies should also better document their study data in the manuscript so that they can be included in future meta-analysis.

Moreover, measures of viral suppression in our study are inconsistent. For example, some studies used a longitudinal measurement for sustained viral suppression, such as “all viral loads in the past year are < 200 copies/ml” [38, 42] or “time to virologic failure” [44]. Some used the most recent or the highest viral load, or the viral load at study completion obtained from medical records [37, 39, 46]. Further, some studies measured undetectable viral load (< 20, 40, or 50 copies/ml) while others used a threshold of 200 copies/ml. The aforementioned results in the subgroup analysis showed influence of the outcome measurement. Similarly, different scales were used to measure depression across the studies, but harmonization of the depression outcome were not conducted due to the lack of granularity of the data. These inconsistencies might have compromised the strength of the current study findings. Nevertheless, the variance in HIV viral suppression measurement indicates a need for further investigation regarding appropriate data collection tools. To examine the long-term effects of depressive symptoms on PLWH, future studies should focus on measures of sustained viral suppression that are more clinically relevant and combine the viral suppression measure with the CD4 cells measure to increase clinical significance [73].

Additionally, the meta-analysis synthesized results from study-level data only. Therefore, although we calculated the pooled odds ratio of viral suppression among PLWH without depressive symptoms compared to those with depressive symptoms, we are unable to draw a definite conclusion at the individual level. Our findings should be interpreted with caution considering that publication bias exists and studies with insignificant results might not have been published. Not including any literature could also have limited the scope of our findings. Our findings might have overestimated the association between non-depression and HIV viral suppression. Lastly, despite a comprehensive search, our strategy might have failed to identify all eligible studies. To mitigate this consequence, we contacted study authors and successfully obtained additional data for two relevant studies.

This systematic review and meta-analysis contributes to the growing body of literature regarding depressive symptoms and HIV viral suppression among PLWH. Our findings suggest the potential benefits of depression intervention on improving HIV viral suppression among PLWH in the HIV care continuum. Further research is needed to address the health disparities in gender and sexual minority groups, explore the needs of diverse and high-risk populations, and improve measurement consistency. Further evaluation of the effectiveness and cost-effectiveness of depression screening and intervention are warranted to guide resource allocation, clinical practice, and mental health program integration into HIV management.

## Appendix: Data extraction and quality assessment processes

To increase the consistency of the extracted data between reviewers, the two primary reviewers participated in a training session to go through detailed instructions and several examples on filling in the data fields in a data extraction table. During the independent data extraction process, the two primary reviewers met every week to compare their data extraction results and discuss any inconsistencies in the data fields, which usually involved discrepancies in statistics or the amount of details being extracted for a specific field. When inconsistencies were found in the statistics extracted, both reviewers would review the original article together and verify the correct statistics before finalizing the data field. When inconsistencies were found in the qualitative details extracted, the primary reviewers would also review the original article together and discuss a way to summarize the details to be inclusive of all necessary information.

For the quality assessment process, one primary analyst was responsible for going through each of the 16 included articles and filling in the scoring fields of the MINORS scale. Another analyst performed an additional round of review of the articles with the information from the MINORS scale and initiated a discussion between analysts if they disagreed upon a specific field of the scoring. As the items in the MINORS scale were clearly described, no significant disagreement arose from this process. The only disagreement was in the item of ‘Endpoints appropriate to the aim of the study’ for two articles, which eventually got resolved through consensus reached between the reviewers after discussion.
